# OLA1 promotes colorectal cancer tumorigenesis by activation of HIF1α/CA9 axis

**DOI:** 10.1186/s12885-022-09508-1

**Published:** 2022-04-19

**Authors:** Yue Liu, Xiang-Xing Kong, Jin-Jie He, Yan-Bo Xu, Jian-Kun Zhang, Lu-Yang Zou, Ke-Feng Ding, Dong Xu

**Affiliations:** 1 Key Laboratory of Cancer Prevention and Intervention, Ministry of Education, Department of Colorectal Surgery and Oncology, The Second Affiliated Hospital, Zhejiang University School of Medicine, Zhejiang, Hangzhou China; 2 Cancer Center, Zhejiang University, Zhejiang, Hangzhou China

**Keywords:** Colorectal cancer, Tumorigenesis, OLA1, HIF 1α, CA9

## Abstract

**Background:**

Obg-like ATPase 1 (OLA1) is a highly conserved GTPase, which was over expressed in a variety of malignant tumors, but its role in colorectal cancer (CRC) was poorly studied.

**Patients and methods:**

Three public CRC gene databases were applied for OLA1 mRNA expression detection. The clinical data of 111 CRC patients were retrospectively collected from the Second Affiliated Hospital of Zhejiang University (SAHZU) for OLA1 protein expression and Kaplan-Meier Survival analysis. OLA1 stably knocked out CRC cell lines were conducted by CRISPR-Cas9 for experiments in vitro and in vivo.

**Results:**

OLA1 was highly expressed in 84% CRC compared to matched surrounding tissues. Patients with OLA1 high expression had a significantly lower 5-year survival rate (47%) than those with OLA1 low expression (75%). OLA1 high expression was an independent factor of poor prognosis in CRC patients. OLA1-KO CRC cell lines showed lower ability of growth and tumorigenesis in vitro and in vivo. By mRNA sequence analysis, we found 113 differential express genes in OLA1-KO cell lines, of which 63 were hypoxic related. HIF1α was a key molecule in hypoxic regulation. Further molecular mechanisms showed HIF1α /CA9 mRNA and/or protein levels were heavily downregulated in OLA1-KO cell lines, which could explain the impaired tumorigenesis. According to previous studies, HIF1α was a downstream gene of GSK3β, we verified GSK3β was over-activated in OLA1-KO cell lines.

**Conclusion:**

OLA1 was a new gene that was associated with carcinogenesis and poor outcomes in CRC by activation of HIF1α/CA9 axis, which may be interpreted by GSK3β.

**Supplementary Information:**

The online version contains supplementary material available at 10.1186/s12885-022-09508-1.

## Text

### Introduction

Colorectal cancer (CRC) is one of the most frequent gastrointestinal malignant tumors and is also the third leading cause of cancer-associated death worldwide [1, 2]. Various genes involved in the development of cancer pathogenesis provide novel avenues to understand the underlying molecular mechanisms that contribute to the development of CRC [3]. However, the effect of genes on the tumorigenesis of CRC is still being elucidated.

OLA1 belongs to the translation-factor (TRAFAC) related class, Obg family, and YchF subfamily of *P*-loop GTPases [4, 5]. The YchF/OLA1 proteins are highly conserved from bacteria to humans. Unlike other Obg family members, OLA1 possess both GTPase and ATPase activities [6, 7]. We have previously reported that mouse embryos lacking OLA1 have immature and small organs at birth and in the later growth and development. It probably indicates its potential role in tumor development [8]. As reported, OLA1 was over expressed in a variety of malignant tumors including CRC, and was an important gene in regulating the growth and metastases in lung cancer and breast cancer [9–11]. However, its role in CRC was poorly studied.

In solid tumors, hypoxia is a prevalent occurrence. Hypoxia-inducible factor 1 (HIF-1) is required for regulating the cellular response to hypoxia by activating hundreds of genes through transcription [12].

HIF-1 is a heterodimer comprised mainly of two subunits called HIF-1α and HIF-1β. The HIF-1α subunit is activated by hypoxia signals and forms a functional HIF-1 with 1β whose transcription factor specifically recognizes hypoxia response elements in the target gene promotors, resulting in transcriptional activation, whereas the HIF-1β subunit is persistently produced in cells and serves a structural function. Cancer cells generate HIF-1 dramatically increased in hypoxic circumstances, promoting anaerobic glycolysis and angiogenesis, which leads to cell proliferation, apoptosis and invasive phenotype [13, 14]. CA9 is one of the target genes of HIF1α [15, 16] which is frequently over expressed in various of malignancies. CA9 appears to be involved in tumor growth and metastatic dissemination in vitro and clinical studies, and overexpression of CA9 is linked to a poor prognosis in a variety of cancers [17, 18]. In CRC, CA9 is proved to promote tumor growth and necrosis in vivo [19].

In the study, we found that OLA1 was highly expressed in most CRC samples by immunohistochemistry, which was associated with poor prognosis. To explore the molecular role of OLA1 in CRC, we constructed two OLA1-KO CRC cell lines Hct116 and Lovo, deprivation of OLA1 led to lower ability of growth and tumorigenesis in vitro and in vivo. By mRNA sequence analysis, we found 113 differential expression genes in OLA1-KO and control cell lines, 63 of which were hypoxia-related. HIF1α was a key molecule in hypoxic regulation. Further molecular mechanisms showed HIF1α /CA9 mRNA and/or protein levels were heavily downregulated in OLA1-KO cell lines, which could explain the impaired tumorigenesis.

## Materials and methods

### Sampling

Public database: Three independent public CRC gene databases, TCGA-RNAseq dataset (*n* = 50), GSE31737 (*n* = 40) and GDS4382 (*n* = 17), were applied for OLA1 mRNA expression detection. Clinical cohort: 111 patients from the Second Affiliated Hospital of Zhejiang University (SAHZU) were enrolled. All patients were confirmed CRC by histopathological examination and had a follow-up over 5 years. Primary tumor specimens and matched adjacent non-tumorous tissues were collected for further OLA1 immunohistochemistry (IHC). All human materials were obtained with informed consent and the study was approved by the Ethical Review Committee of SAHZU (2021-LYS-0689).

### Clinical data collection

The expression quantity of OLA1 mRNA, normalized by log2, was extracted from TCGA-RNA dataset, GSE31737 and GDS4382. The relative expression between CRC and paired adjacent normal tissue was analyzed within the three datasets respectively. Both primary tumor and adjacent non-tumorous tissues from SAHZU were performed OLA1 IHC. The patients were divided into OLA1 high (IHC-score: 2 or 3) group and OLA1 low (IHC-score: 0 or 1) group by IHC-score according to DAB dye depth.

### Cell culture

Human colon cancer cell lines HCT116 and Lovo were purchased from American Type Culture Collection (ATCC). Cell lines were cultured under standard conditions in 1640 medium (Gibco) with 10% fetal bovine serum (FBS, Hyclone), 10 units/ml penicillin, and 10 mg/ml streptomycin at 37 °C and 5% CO2 in a humidified incubator (Thermo Scientific) and regularly tested for mycoplasma contamination.

### Establishment of OLA1-KO CRC cell line by CRISPR/Cas9

Stable OLA1-KO cell lines (HCT116-KO, Lovo-KO) were established using CRISPR/Cas9. Designed CRISPR/Cas9 target OLA1 gRNA OLA1-gRNA1: ACGTTCACCTACCCAACATTTGG and OLA1-gRNA2: ATTTCAGATGCCCCCTAAAAAGG, conducted the knockout vector and transfected into Hct116 and Lovo cells by electro-transfection. Screening monoclonal cells by PCR. The knockout efficiency was verified by western blot analysis.

### In vitro cell proliferation, colony formation assays

The cell proliferation assay was measured using the Cell Counting Kit-8 (CCK-8) (Dojindo, Kyushu, Japan). Experimental and control cells were both placed into the 96-well plate with a density of 1000 cells/well, and 10 μl of CCK-8 was added into 90 μl of the culture medium per well. After incubating at 37 °C for 2h, and the density was measured at 450 nm.

For the colony formation assay, experimental and control cells were plated into a 6-well plate with a density of 1000 cells/well and 500 cells/well respectively. Cells were incubated at 37 °C for 2 weeks. The colonies were fixed and stained using 20% methanol and 0.1% crystal violet, and the number of colonies (> 50 cells) was counted. All assays were replicated three times.

### In vivo assays for tumor formation

This was conducted by subcutaneous tumor formation in nude mouse. 1 × 10^6^ experimental and control colon cancer cells were suspended in 100 μl PBS and subcutaneously injected into nude mouse (female nude mouse, 10 per group). The tumor sizes were measured every 3 days as soon as the tumors were measurable. The mice were sacrificed after 28 days and the tumors were harvested for further analysis.

### mRNA sequence analysis

Total mRNA isolated from OLA1-KO and control cells. DNase I was used to digest double-stranded and single-stranded DNA in total RNA, then, magnetic beads were purified to recover the reaction products, RNase H or Ribo-Zero method (Human, mouse, plants) (Illumina, USA) was used to remove the rRNA. Purified mRNA from previous steps was fragmented into small pieces with fragment buffer at the appropriate temperature. Then, the first-strand cDNA was generated in First Strand Reaction System by PCR, and the second-strand cDNA was generated as well. The reaction product was purified by magnetic beads, afterwards, A-Tailing Mix and RNA Index Adapters were added by incubating to carry out end repair. The cDNA fragments with adapters were amplified by PCR, and the products were purified by Ampure XP Beads. Library was validating on the Agilent Technologies 2100 bioanalyzer for quality control. The double-stranded PCR products above were heated denatured and circularized by the splint oligo sequence. The single-stranded circle DNA (ssCir DNA) was formatted as the final library. The final library was amplified with phi29 (thermos Fisher Scientific, MA, USA) to make DNA nanoball (DNB) which had more than 300 copies of one molecular, DNB were loaded into the patterned nanoarray and single end 50 bases reads were generated on BGISEQ500 platform (BGI-Shenzhen, China) [20].

### Western blot

Equal amounts of cellular protein were separated by sodium dodecyl sulfate-polyacrylamide gel electrophoresis (SDS-PAGE) and transferred onto polyvinylidene fluoride (PVDF) membranes (Bio-Rad, Hercules, CA). The membrane was blocked with 5% non-fat milk for 1 h and incubated with primary and secondary antibodies. Immunoreactivity was visualized using chemiluminescence ECL reagents (Pierce, Rockford, IL). The densitometry analysis was measured by Image-Pro Plus 6.0 (Media Cybernetics). All antibodies used in these studies were as follows: anti-OLA1, (Sigma-Aldrich,1:500), anti-GSK3β, (cell signaling technology,1:1000), anti-P-GSK3β, (cell signaling technology,1:1000), anti-CA9(sigma-Aldrich,1:500), anti-GAPDH (cell signaling technology,1:5000), anti-STAT3, (cell signaling technology,1:1000), and anti-rabbit/mouse IgG (cell signaling technology,1:5000), peroxidase-linked secondary antibody. The images were collected by Azure multifunctional molecular imaging system (AZURE600) and scanner.

### IHC staining

IHC was performed for OLA1 on 90 paired formalin-fixed, paraffin-embedded samples and 111 formalin-fixed, paraffin-embedded colorectal cancer samples. In a nutshell, sections were deparaffinized, rehydrated and pretreated in a pressure cooker in 1 mM EDTA buffer pH 8.0. Slides were then incubated with a polyclonal antibody against OLA1, diluted 1:800 (Sigma, #HPA027524), CA9, diluted 1:500 (sigma-Aldrich), HIF1α, diluted 1:500 (cell signaling technology), Ki67, diluted 1:1000 (cell signaling technology) at 37 °C for 2 h. Slides were rinsed three times with PBS containing 0.05% Tween20 and developed with the EnVision-System (DAKO, #K4003) according to the manufacturer’s protocol. Finally, they were counterstained with hematoxylin, diluted 1:10(Merck; #1.09249.0500) for 60–90 s. Two researchers masked sections and analyzed them independently. The image of IHC was collected by light microscope.

### Real-time quantitative reverse transcription-polymerase chain reaction

The Qiagen RNeasy Mini kit was used to extract total RNA from the cells according to the manufacturer’s guidelines. We evaluated the quality and quantity of the RNA by a NanoDrop 1000 spectrophotometer (Thermo Scientific, Pittsburgh, PA, USA). The quality and amount of the RNA was assessed by a NanoDrop 1000 spectrophotometer (Thermo Scientific, Pittsburgh, PA, USA). The PrimeScript™ II 1st Strand cDNA Synthesis Kit (Takara Biotechnology, Dalian, China) was used to generate the cDNA we needed. The Step-One-Plus Real-Time PCR System (Life Technologies, Foster, CA, USA) was utilized for quantitative real-time RT-PCR (qRT-PCR), whose data were analyzed via Step-One software V2.1. Genetic expression was measured with the TaqMan test system and the following keys and probes: ola1 forward: 5′-TGGACAAGTATGACCCAGGT-3′, reverse: 5′-GCTGCAAACCCAGCCTTAATG-3′; ca9 forward: 5′- TACAGCTGAACTTCCGAGCG-3′, reverse: 5′- CTAGGCTCCAGTCTCGGCTA-3′; gapdh forward: 5′- CATGAGAAGTATGACAACAGCCT-3′, reverse: 5′- AGTCCTTCCACGATACCAAAGT-3′.

### Animal and embryo studies

Generation of mouse strains carrying *Ola1* null mutations has been described previously [8]. Time matings were set up by housing a single male with one or two females. At day 18.5 embryonic age, pregnant females were euthanized using CO_2_ asphyxiation and the embryos were dissected. Thermo Scientific T-PER tissue protein extraction (No.78510) and Thermo Scientific Halt Protease Inhibitor Cocktail was used. Scissor 1 g of soft tissues of mouse embryo (excluded skeletal, skin, brain, lung, liver), add 30ul T-PER reagent with cocktail and homogenize, lysis on ice for 45 to 60 min, then centrifuge the sample at 10,000×g for 5 min to pellet tissue debris, finally, Collect supernatant for western blot.

### Statistical analysis

All of the results came from at least three separate experiments. For comparisons between two groups, the data were reported as mean ± SD and the Student’s t test was utilized. The population results are shown as percentages, medians, and 95% confidence intervals (CI). The population results are shown as percentage, medians, and 95% confidence interval (CI). The Kaplan-Meier estimator technique was used to produce overall survival (OS) curves. The univariate and multivariate Cox’s proportional hazards models were used to investigate prognostic factors. A statistically significant difference was defined as *P* < 0.05. The ratio of the relative expression of the indicated gene from the tumor patients was plotted and applied with the spearman correlation test. A *p* value of 0.05 was considered statistically significant. In the graphed data *, **, and *** denote *p* values of < 0.05, 0.01 and 0.001, respectively. All of the statistical analyses were performed by using SPSS18.0.

## Results

### OLA1 was upregulated in CRC and linked to a poor prognosis

**Fig. 1:**
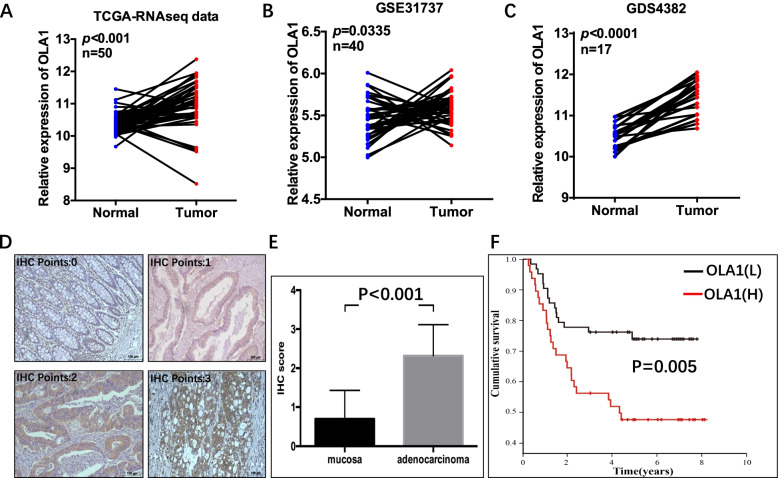
Overexpression of OLA1 in clinical CRC samples. **A**-**E** Relative expression of OLA1 in paired colorectal cancer and matched normal tissue samples from **A** TCGA-RNAseq dataset, **B** GEO GSE31737 dataset, **C** GDS4382 dataset and **D**, **E** the tissue bank in our laboratory. Data are presented as mean ± SD (*n* = 90); statistical significance was assessed by paired t-test. **F** Kaplan-Meier plots of CRC patients with different OLA1 IHC score OLA1 expression in our hospital, OLA1 (H) defines as IHC score 2 and 3, and OLA1 (L) were IHC score 0 and 1. CRC, colorectal cancer; TCGA, The Cancer Genome Atlas

OLA1 gene copy number was dramatically increased in CRC, according to the Cancer Genome Atlas (TCGA) and the GEO database (Fig. 1A-C). However, the higher OLA1 copy numbers were identified in all stages of CRC and were unconnected with tumor stage. In the samples from the TCGA dataset (Fig. 1A), GEO GSE31737 dataset (Fig. 1B), and GDS4382 dataset (Fig. 1C), the expression level of OLA1 in matched-paired CRC and normal tissue samples was investigated. The results demonstrated tumor tissue samples had a considerable up-regulation of OLA1 in these datasets.

To validate the role of OLA1 in colorectal cancer in these databases, OLA1 expression was analyzed in CRC tissues by IHC (Fig. 1D). Results showed that 84% (76/90) primary CRC tumors had higher OLA1 expression (Fig. 1E) compared to the matched surrounding tissues. However, there was no significant correlation between OLA1 expression and other clinicopathological variables, such as patient gender, age, tumor size, and TNM stage (*P* > 0.05, Table 1). Patients with high OLA1 expression showed a considerably poorer survival rate than those with low OLA1 expression, according to a Kaplan–Meier analysis (Fig. 1F, Table 2). CRC patients with high OLA1 expression had a 5-year survival rate of 47%, which was substantially lower than those with low OLA1 expression (75%). To assess whether OLA1 high expression was an independent prognostic factor, all variables that may affect the OS were included in a Cox’s proportional hazards model (Table 3). Independent prognostic factors were: T stage [hazard ratio (HR) 2.092; 95% CI (1.090–4.031); *P* = 0.026], N stage [HR 1.383; 95% CI (1.018–1.878); *P* = 0.038], M stage [HR 4.407; 95% CI (2.033–9.552); *P* < 0.001], and OLA1 high expression [HR 2.616; 95% CI (1.328–5.153); *P* = 0.005].

**Table 1:** Baseline of patients with different OLA1 expression level

	OLA1 Low (***N*** = 63)	OLA1 High (***N*** = 48)	***p***
**Age**	58.84 ± 14.21	49.56 ± 15.12	0.797
**Gender**			0.497
Male	34(54.0%)	29(60.4%)	
Female	29(46.0%)	19(39.6%)	
**Tumor location**			0.577
Right colon	20(31.7%)	11(22.9%)	
Left colon	13(20.6%)	12(25.0%)	
Rectum	30(47.6%)	24(52.1%)	
**T stage**			0.147
T1	4(6.3%)	3(6.2%)	
T2	16(25.4%)	7(14.6%)	
T3	22(34.9%)	27(56.2%)	
T4	21(33.3%)	11(22.9%)	
**N stage**			0.702
N0	36(57.1%)	22(45.8%)	
N1	14(22.2%)	13(27.1%)	
N2a	4(6.3%)	4(8.3%)	
N2b	9(14.3%)	9(18.8%)	
**M stage**			0.159
M0	57(90.5%)	39(81.2%)	
M1	6(9.5%)	9(18.8%)	
**Tumor size**			0.938
≤ 3 cm	17(27.0%)	14(29.2%)	
3-5 cm	31(49.2%)	22(45.8%)	
>5 cm	15(23.8%)	12(25.0%)	
**Tumor differentiation**			0.425
Poor	4(6.3%)	2(4.2%)	
Moderate	25(39.7%)	25(52.1%)	
Well	24(38.1%)	15(31.2%)	
Mucous	7(11.1%)	6(12.5%)	
Signet ring	3(4.8%)	0(0.0%)	
**Chemotherapy**			0.721
No	44(69.8%)	32(66.7%)	
Yes	19(30.2%)	16(33.3%)

**Table 2:** Univariate analysis of prognostic factors for overall survival

	Patient		Kaplan-Meier, Log-Rank test	
	N	%	5-year survival	***P***-value
**Age (years)**				0.270
≤ 40	13	12	46%	
40–70	76	68	65%	
≥ 70	22	20	64%	
**Gender**				0.181
Male	63	57	70%	
Female	48	43	58%	
**Tumor location**				0.126
Right colon	31	28	53%	
Left colon	25	23	72%	
Rectum	55	50	66%	
**Tumor differentiation**				0.119
Well	6	5	69%	
Moderate	50	45	68%	
Poor	39	35	35%	
mucous	13	12	60%	
Signet ring	3	3	35%	
**Tumor size (cm)**				0.009
≤ 3	31	28	80%	
3–5	53	48	65%	
>5	27	24	44%	
**T stage**				0.000
T1	7	6	100%	
T2	23	21	95%	
T3	49	44	53%	
T4	32	29	45%	
**N stage**				0.000
N0	58	52	82%	
N1	27	24	52%	
N2a	8	7	50%	
N2b	18	16	28%	
**M stage**				0.000
M0	96	86	73%	
M1	15	14	8%	
**Chemotherapy**				0.001
Yes	35	32	42%	
No	76	68	76%	
**OLA1 score**				0.005
0 + 1	63	57	78%	
2 + 3	48	43	48%

**Table 3:** Multivariate analysis for overall survival in colorectal cancer

	Overall survival		
	HR	95% CI	***P***-value
**Gender**	0.717	0.352–1.462	0.360
**Age**	0.833	0.425–1.632	0.594
**OLA1 score**	2.616	1.328–5.153	0.005
**Tumor location**	1.285	0.828–1.994	0.263
**T stage**	2.092	1.090–4.031	0.026
**N stage**	1.383	1.018–1.878	0.038
**M stage**	4.407	2.033–9.552	0.000
**Tumor size**	1.292	0.767–2.177	0.336
**Tumor differentiation**	1.070	0.739–1.548	0.720
**Chemotherapy**	1.390	0.694–2.783	0.353

*HR* Hazard ratio, *CI* confidence interval

In addition, we analyzed the OLA1 protein expression in eight colorectal cancer cell lines by western blot, and the result indicated that OLA1 was high expression in most of these cell lines (Fig. S1). Together, these results clearly showed that OLA1 was overexpressed in CRC tissues and CRC cells, suggesting that OLA1 might play a role in CRC carcinogenesis and development.

### Knockout of OLA1 inhibited growth of CRC cells in vitro

**Fig. 2:**
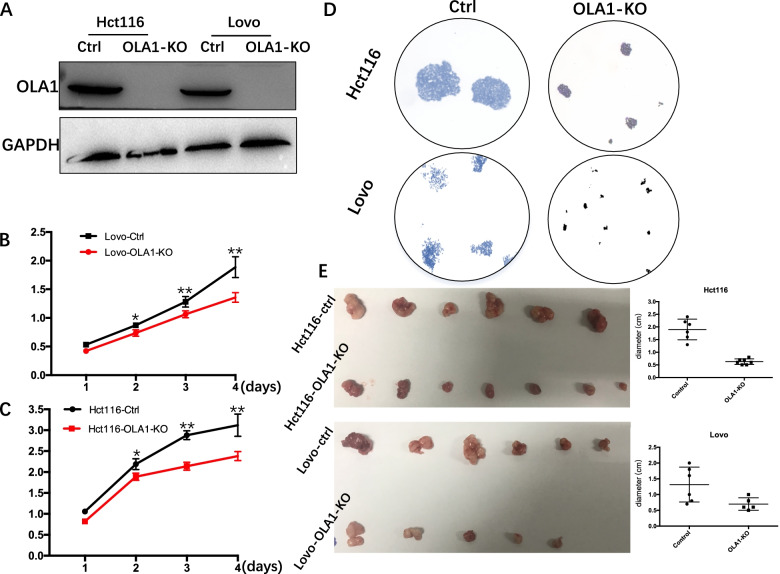
OLA1 knockout inhibits proliferation in vitro and in vivo. **A** Immunoblotting analysis of OLA1 protein level in control and OLA1-KO cell lines (Hct116 and Lovo cells, the blots in this figure were cut prior to hybridization with antibody GAPDH and OLA1); **B**, **C** CCK-8 assay for investigating proliferation potential of Ctrl and OLA1-KO cell lines, (B) Hct116 and (C) Lovo. **D** Colony formation assay for investigating colony formation ability of Ctrl and OLA1-KO cell lines; **E** Xenograft model of BALB/c-nu/nu female mice with subcutaneous injection Hct116 and Hct116-OLA1-KO cells(left-up), Lovo and Lovo-OLA1-KO cells(left-down), tumor volume when mice were executed in xenograft model (right). Data are presented as mean ± SD; statistical significance was assessed by unpaired t-test. *P < 0.05, **P < 0.01, ***P < 0.001; GAPDH, glyceraldehyde 3-phosphate dehydrogenase; Ctrl, scramble; OLA1-KO, OLA1 knockout

The prevalence of OLA1 upregulation raises an intriguing possibility that OLA1 overexpression may be a cancer-promoting event in CRC. To verify this hypothesis, OLA1 was stably knocked out by CRISPR/Cas9 in Lovo and Hct116 cells expressing high levels of OLA1 protein. The KO efficiency was confirmed by western blotting (Fig. 2A). The CCK8 assays showed that knocking out OLA1 significantly reduced cellular proliferation (Fig. 2B and C). Colony formation assays were also used to investigate the role of OLA1 in cell proliferation (Fig. 2D). The colony formation ability was significantly impaired when these two cells lacked of OLA1. Collectively, these data demonstrated that OLA1 can promote colorectal cancer cell growth in vitro.

### OLA1 promoted the growth of CRC cells in mouse models in vivo

**Fig. 3:**
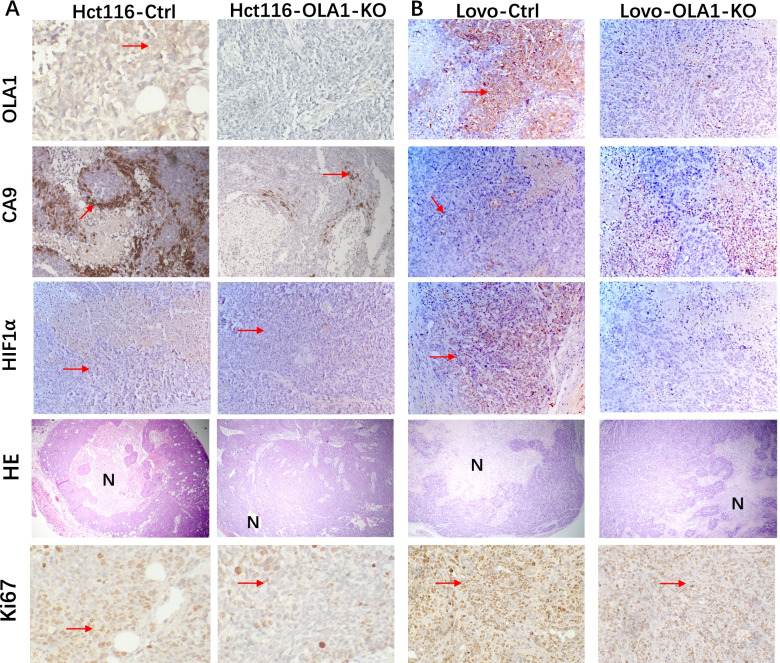
OLA1 expression is associated with increased CA9, necrosis and proliferation in HCT116 and Lovo xenografts. Representative immunohistochemical images of HCT116 (**A**) and Lovo (**B**), control and OLA1-KO xenografts. The expression of OLA1, CA9, HIF1, Ki67, and the fraction of necrosis between the control and OLA1-KO groups were substantially different. The expression staining of OLA1, CA9, HIF-1, and Ki-67 were depicted in dark brown. The dark pink stain in H&E indicates living tissue, while the light pink stain indicates necrosis. SD is represented by the error bars. Necrosis is indicated by the letter N. Positive staining is indicated by red arrows

The nude mice’s flanks were injected with the same number of OLA1-KO and control CRC cells Hct116 and Lovo. Every 3 days, tumor formation was tracked and tumor sizes were measured. Tumors were established on 12 days after implantation, as evidenced by the fact that OLA1-KO cells developed much more slowly than control cells, and tumor sizes in these mice were significantly less than those in control mice (Fig. 2E). IHC also confirmed the extent of OLA1 depletion in the tumors (Fig. 3). To sum up, these findings demonstrated low OLA1 expression were sufficient to suppress CRC cell growth, strongly suggesting that OLA1 acted as a tumor promoter in CRC.

### Modulation of hypoxia-related gene expression by OLA1 in CRC cells

**Fig. 4:**
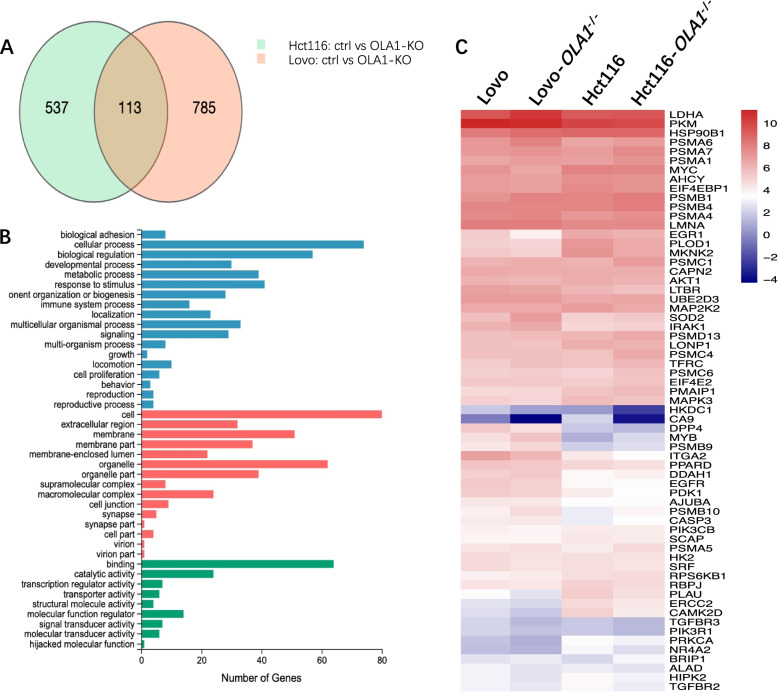
mRNA sequencing in OLA1-KO and Ctrl cell lines. Gene expression was analyzed using the Affymetrix Human Genome U133 Plus 2.0 Array. **A** 113 genes were changed in both pairs of Hct116 and Lovo cell lines. **B** Functional annotations of differentially expressed genes in OLA1-KO and Ctrl cell lines with functions related to key cellular processes, including regulation of cell proliferation, cell invasion, cell cycle, glucose metabolism and formation of nucleosome structure. **C** By pathways on gene-set enrichment analysis, Of the 113 common changed genes, 63 genes were hypoxia-related genes.

In order to clarify the underlying mechanisms of OLA1 in CRC, we performed mRNA sequencing in OLA1-KO and control cells. Biological replicates were 2 pairs of colon cancer cell line Hct116 and Lovo. Gene expression was analyzed using the Affymetrix Human Genome U133 Plus 2.0 Array. The mRNA expression of OLA1 was validated through qRT-PCR. As showed in Fig. 4A, 113 genes were changed in both pairs of cell lines. Functional annotations of these genes indicated that there were a number of differentially expressed genes with functions related to key cellular processes, including regulation of cell proliferation, invasion, cell cycle, glucose metabolism and formation of nucleosome structure (Fig. 4B). Interestingly, 63 genes were hypoxia-related genes among these 113 common changed genes (Fig. 4C). Overall, these data suggested that OLA1 promoted the tumorigenesis of CRC by upregulating expression of proliferation, invasion and cell cycle related genes.

### CA9 and HIF1α were downregulated in OLA1-KO CRC cell lines

HIF1α, which was a key molecule in hypoxic regulation, was examined by IHC in OLA1-KO xenografts, and the result showed that HIF1α was downregulated in OLA1-KO groups. Meanwhile, Ki67 and necrosis were also decreased in OLA1-KO groups (Fig. 3). CA9 was a target gene of HIF1α, and was the most upregulated gene in the mRNA sequence. By qRT-PCR, western blot and IHC, we found CA9 level was heavily downregulated in OLA1-KO cell lines both in mRNA and protein level. (Fig.3, and 5A-B). These results indicated that OLA1 depletion inactivated HIF1α signaling which caused growth retardation.

**Fig. 5:**
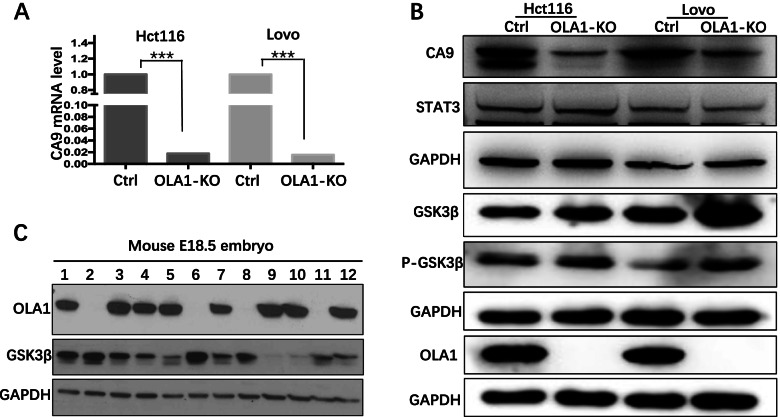
The differential expression of HIF1α and CA9 in Ctrl and OLA1-KO CRC cell lines. **A** CA9 mRNA expression was heavily downregulated in OLA1-KO CRC cell lines by real-time qRT-PCR (*P* < 0.001). **B** CA9 protein was downregulated and GSK3β was upregulated in OLA1-KO CRC cell lines by western blot (the blots in this figure were cut prior to hybridization with antibody GSK3β, P- GSK3β, GAPDH and OLA1). **C** In OLA1-KO mouse E18.5 embryo tissues, GSK3β was upregulated compared with the control tissues. The number 1 to 12 represent 12 different tissues from 12 embryos. Number 1 to 7, 8 to 9, and 10 to 12 from 3 mothers separately

### Overactivation of GSK3β in OLA1-KO cell lines inhibited HIF1α signaling

Ola1^−/−^ knockout mice had been generated in our previous study, a large majority of which were found dead on the day of birth. Ola1^−/−^ embryos were significantly smaller in size than their Ola1^+/+^ and Ola1^+/−^ controls as measured on E18.5. We collected the tissue and extracted protein of E18.5, GSK3β expression was much heavier in Ola1^−/−^ mice than the controls (Fig. 5C). Further, in our study, we validated the GSK3β activity in control and OLA1-KO CRC cell lines, and GSK3β was over-activated in OLA1-KO cell lines (Fig. 5B). Our previous study showed that OLA1 directly interacted with GSK3β and inhibited its activity [21, 22], which is also proved by other study [23, 24]. Meanwhile, GSK3β can phosphorylate HIF1α and facilitate its degradation [25], which can explain why HIF1α decreased in OLA1-KO CRC cell lines.

## Discussion

OLA1 is a member of the GTPase protein family; unlike other members, it possesses both GTPase and ATPase activities, and can bind and hydrolyze ATP more efficiently than GTP. OLA1 participates in cell proliferation, oxidative response, protein synthesis and tumorigenesis [6]. According to previous studies, OLA1 can interact with BRCA1 to regulate the centrosome number, which promotes the proliferation of breast cancer cells [9]; OLA1 deficiency causes p21 accumulation which leads to stunted growth and frequent perinatal lethality in mouse embryonic development [8]. All these data indicates that the function of OLA1 is tightly related to growth and proliferation.

In the study, we found that OLA1 was an important gene in CRC. The expression of OLA1 was obviously elevated in GEO and TCGA databases and also the database in our own center. The result was in accordance with previous studies [10]. Meanwhile, the study first illustrated that elevated expression of OLA1 indicated poor prognosis in CRC. By Cox regression model, OLA1 high expression was the second highest risk factor of CRC, only to distant metastases. The clinical significance of OLA1 gave us more confidence to explore its molecular mechanisms in CRC, like BRAF and KRAS [26, 27]. Further experiments confirmed OLA1 deficiency injured the ability of proliferation, colony formation and tumorigenesis in CRC cell lines, which were accordance with the clinical data.

To explore the molecular mechanism of OLA1 in CRC, mRNA sequence was conducted in OLA1-KO CRC and control cell lines. In these two pairs of colon cancer cell lines (Hct116 and Lovo), we found CA9 mRNA decreased in both Hct116 and Lovo OLA1-KO cell lines.

CA9 is an important gene in carcinogenesis. In CRC samples studied by cDNA microarray, CA9 was found to be the most upregulated gene [28]. In CRC, CA9 showed heavier expression in carcinomas than in benign lesions [29, 30]. The ratio of CA9 positive cells in the normal intestinal epithelia is as low as 10% [31]. The expression pattern of CA9 was just in accordance with OLA1 in CRC.

CA9 is also a marker for poor clinical outcome in most cancer types [10, 19, 28, 30]. The strong staining intensity of CA9 is an adverse prognostic factor in rectal cancer [32]. In CRC cell lines, CA9 promotes tumor growth and necrosis in vivo [19]. Tumor necrosis is a promising prognostic factor in colorectal cancer [33]. We observed the same phenomenon in our study. In xenograft, controlled tumors showed a gradient of CA9 expression with highest levels adjacent to frank necrosis, while in OLA1-KO tumors, CA9, necrosis and Ki67 decreased obviously. High expression of OLA1 indicated poor prognosis maybe related with more necrosis and Ki67 caused by increased CA9.

CA9 is mainly regulated by HIF1α, where the HRE/HIF1α–binding site is located at 3 of 10 position in the promoter region of this gene [15]. HIF1α also contributes to intestinal tumorigenesis [34]. We tested HIF1α protein by western blot in OLA1 control and KO cell lines and by IHC in OLA1 knockout nude mice subcutaneous tumors. We failed to detect it by western blot, which might ascribe to the short life time of HIF-1α under normoxic cell culture conditions [35–38]. But the IHC results confirmed HIF1α expression decreased in OLA1-KO groups which was in accordance with CA9, Ki67 and necrosis. At the same time, we found an interesting phenomenon. In Hct116 cell lines, HIF1α increased less than CA9 while in Lovo cell lines, HIF1α increased more than CA9. This was also reported by Solmaz Sobhanifar et al. [35]. This can be ascribed to long lifetime of CA9 and short lifetime of HIF1α. Since CA9 is a target gene of HIF1α, maybe there is a feedback loop between CA9 and HIF1α.

However, our study did not illustrate how OLA1 influenced HIF1α/CA9 axis. The possible mechanism was the activity of HIF1α was regulated by a variety of genes including GSK3β, and GSK3β can directly interact with OLA1 [39]. OLA1 may upregulate HIF1α expression by GSK3β which needs further study.

This study proposed the possibility that OLA1 could be a new gene that it was associated with carcinogenesis and poor outcomes in CRC by facilitating HIF1α/ CA9 signaling. This process may be interpreted by GSK3β.

## Supplementary Information

**Additional file 1.**

## Data Availability

The sequence data generated in this study are publicly available in The Cancer Genome Atlas(TCGA) -RNAseq dataset, in Gene Expression Omnibus (GEO) at GSE31737 and GDS4382. Further inquiries can be directed to the corresponding author.
