# Supplementary material for: OLA1 promotes colorectal cancer tumorigenesis by activation of HIF1α/CA9 axis
Source: BMC Cancer. 2022 Apr 19;22:424. doi: 10.1186/s12885-022-09508-1 (PMC9020043; doi:10.1186/s12885-022-09508-1)
Supplement: Supplementary file 1 — Additional file 1. [file 12885_2022_9508_MOESM1_ESM.zip › 2nd-wb.pptx]

## Slide 1
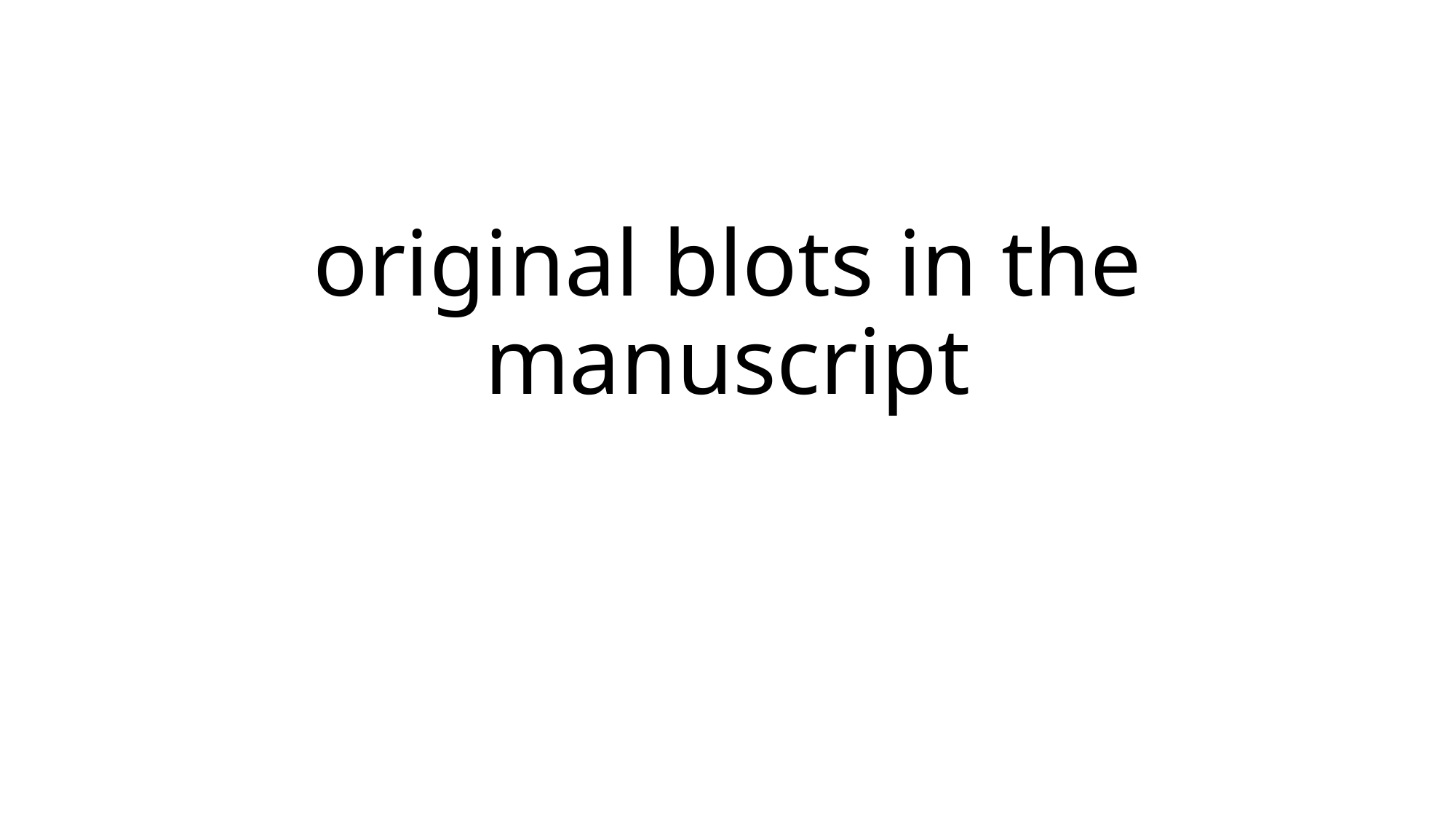

# original blots in the manuscript

## Slide 2
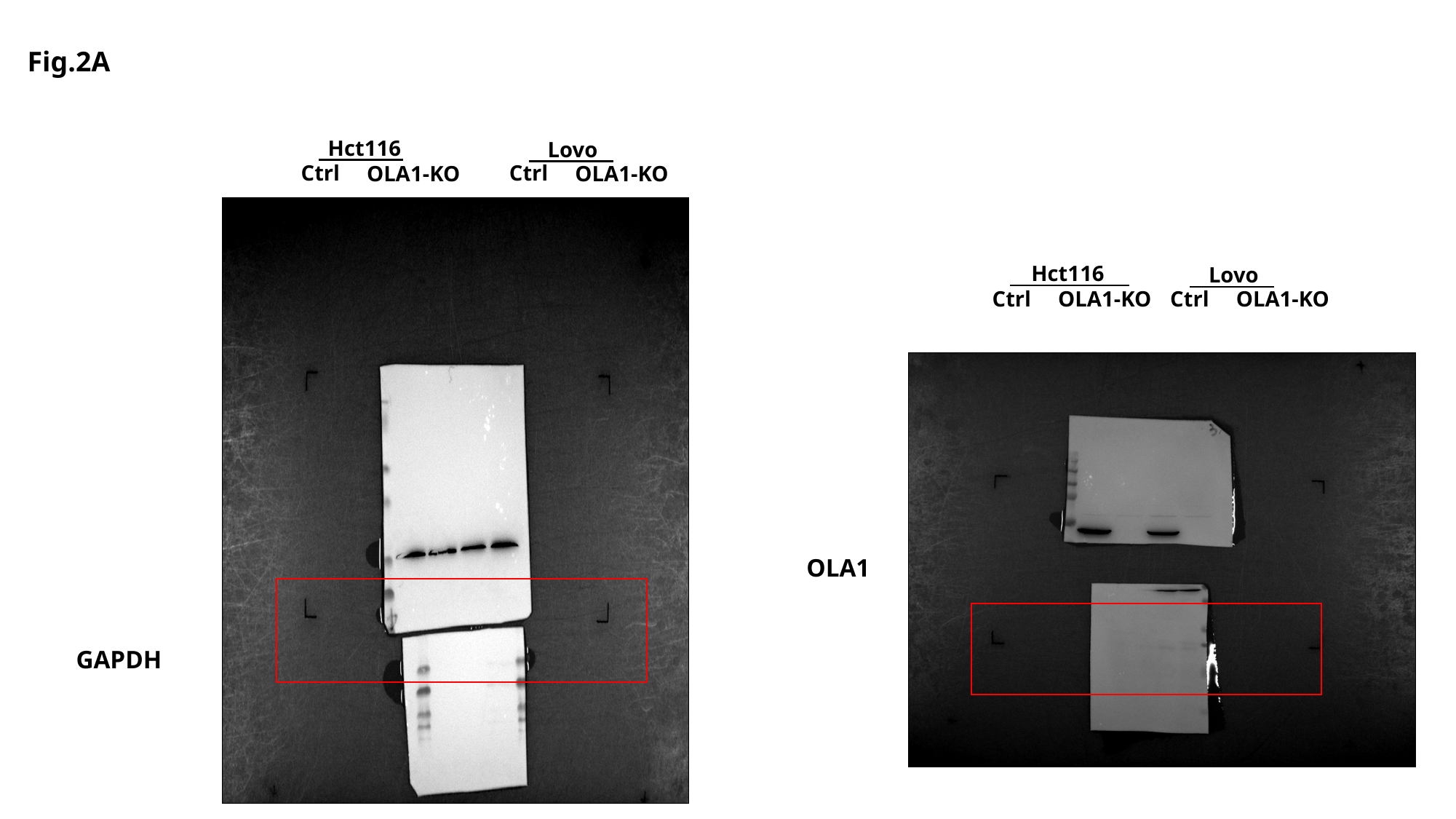

Fig.2A
Hct116
Lovo
Ctrl
Ctrl
OLA1-KO
OLA1-KO
Hct116
Lovo
Ctrl
Ctrl
OLA1-KO
OLA1-KO
OLA1
GAPDH

## Slide 3
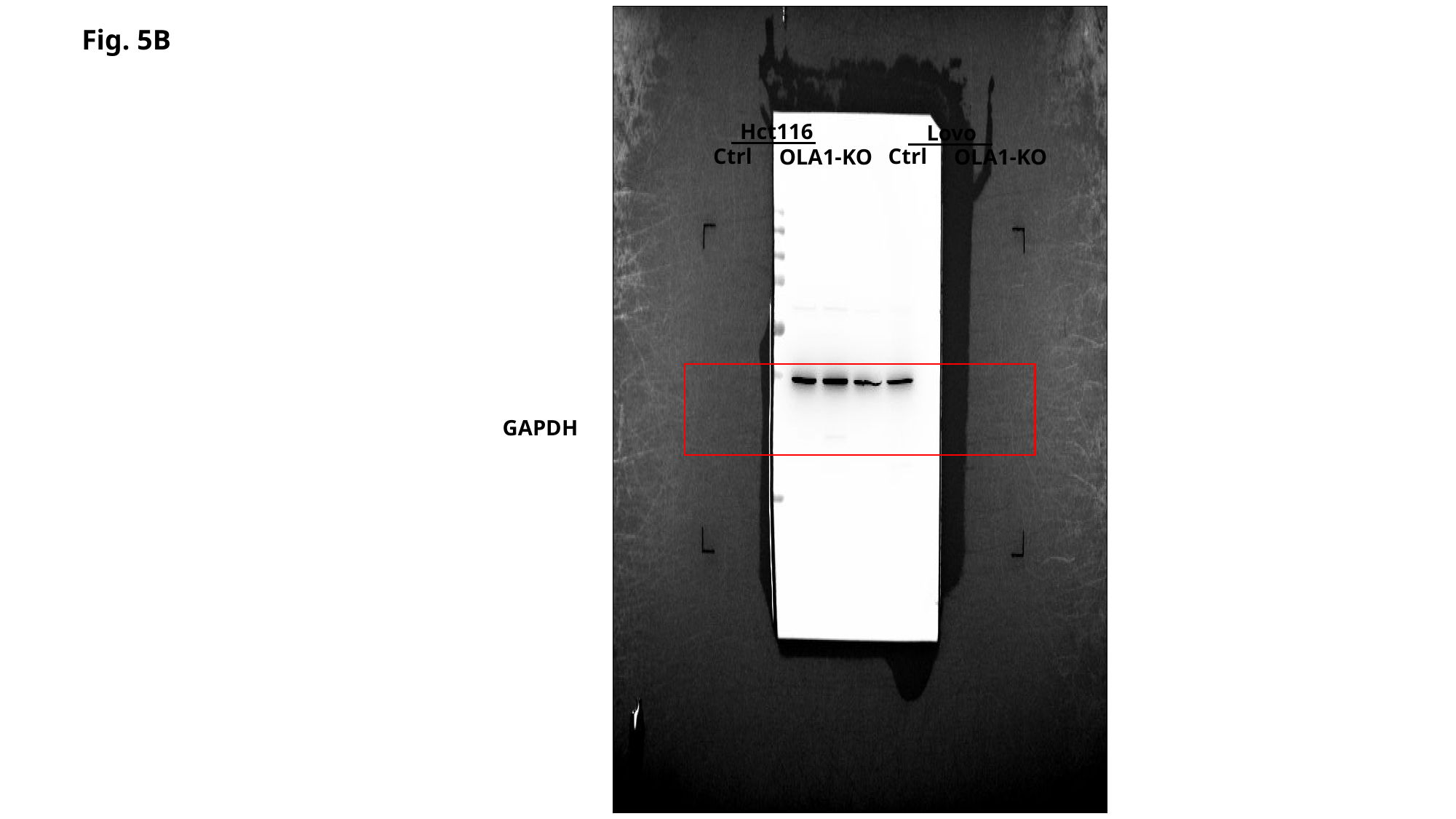

Fig. 5B
Hct116
Lovo
Ctrl
Ctrl
OLA1-KO
OLA1-KO
GAPDH

## Slide 4
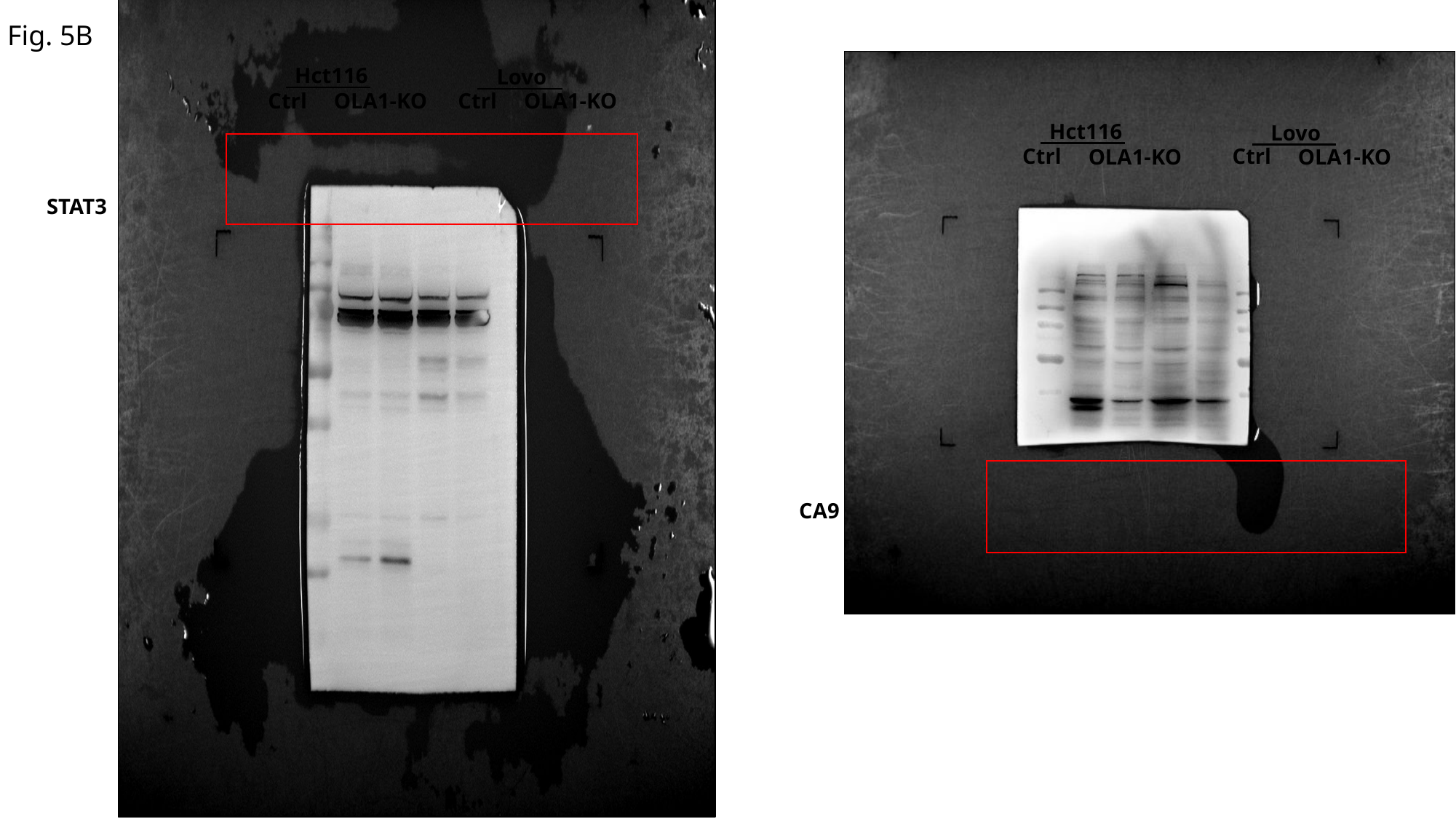

Fig. 5B
Hct116
Lovo
Ctrl
Ctrl
OLA1-KO
OLA1-KO
Hct116
Lovo
Ctrl
Ctrl
OLA1-KO
OLA1-KO
STAT3
CA9

## Slide 5
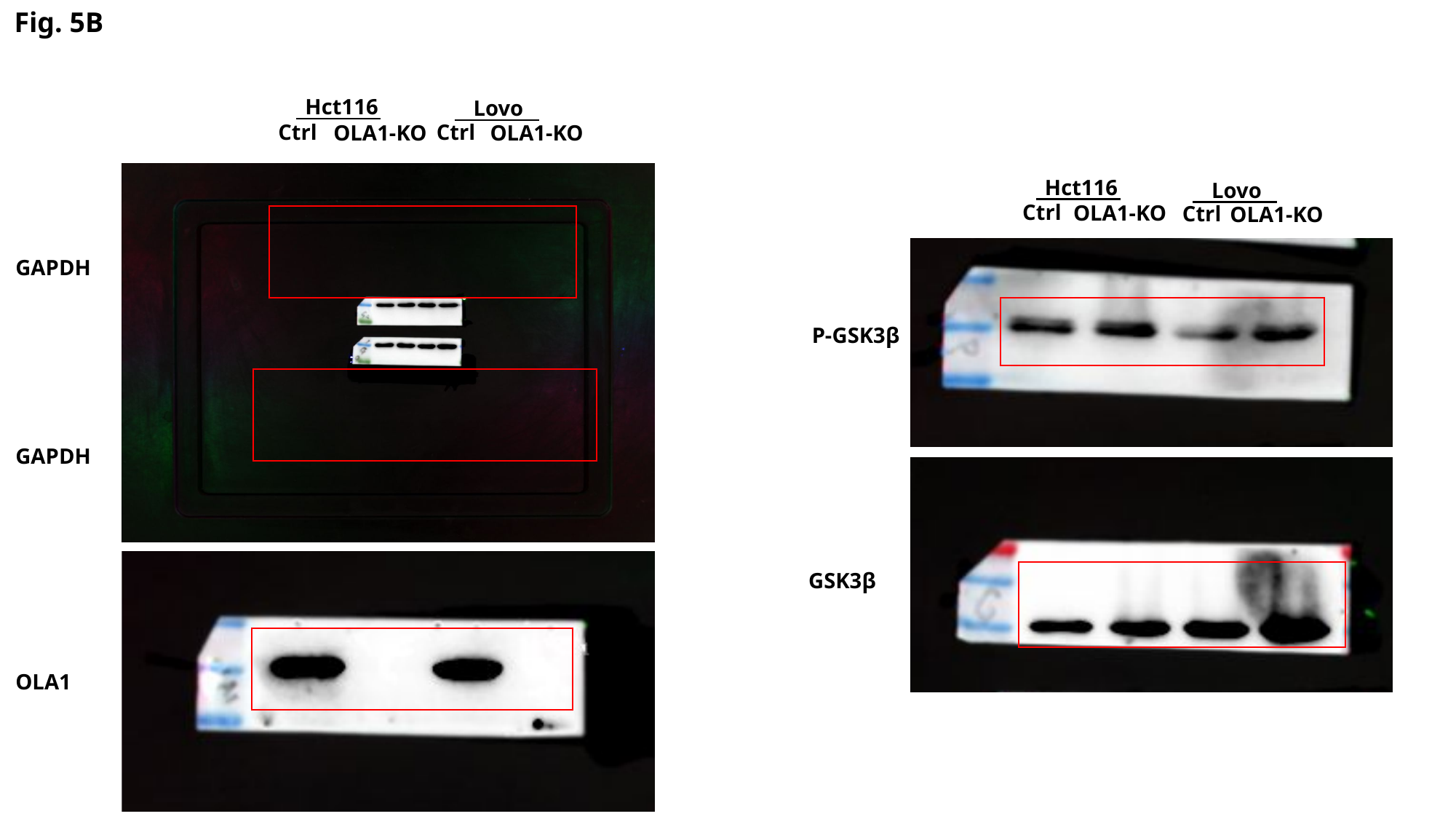

Fig. 5B
Hct116
Lovo
Ctrl
Ctrl
OLA1-KO
OLA1-KO
Hct116
Lovo
Ctrl
OLA1-KO
Ctrl
OLA1-KO
GAPDH
P-GSK3β
GAPDH
GSK3β
OLA1

## Slide 6
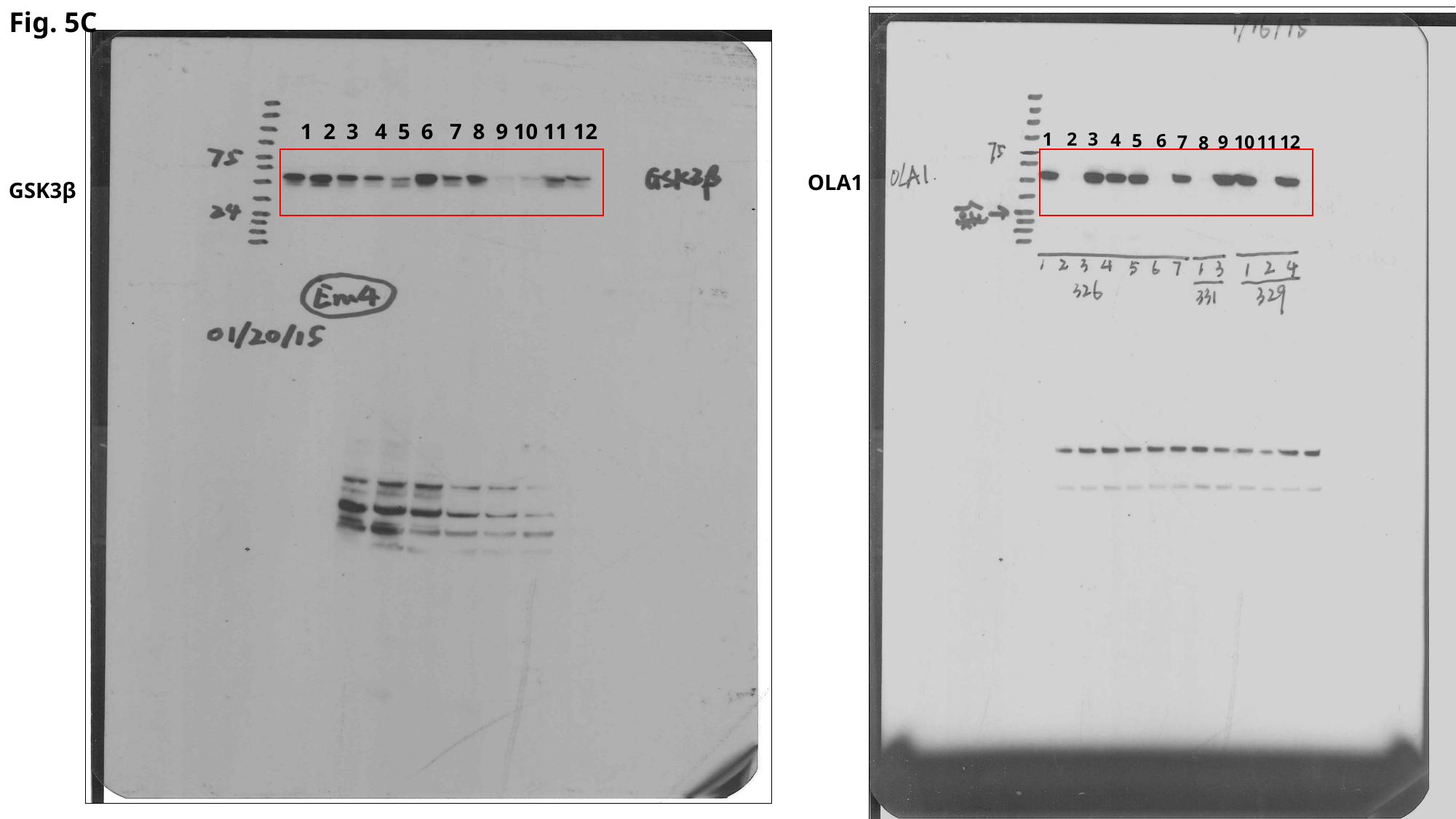

Fig. 5C
1 2 3 4 5 6 7 8 9 10 11 12
2
1
3
4
5
6
11
12
10
7
9
8
OLA1
GSK3β

## Slide 7
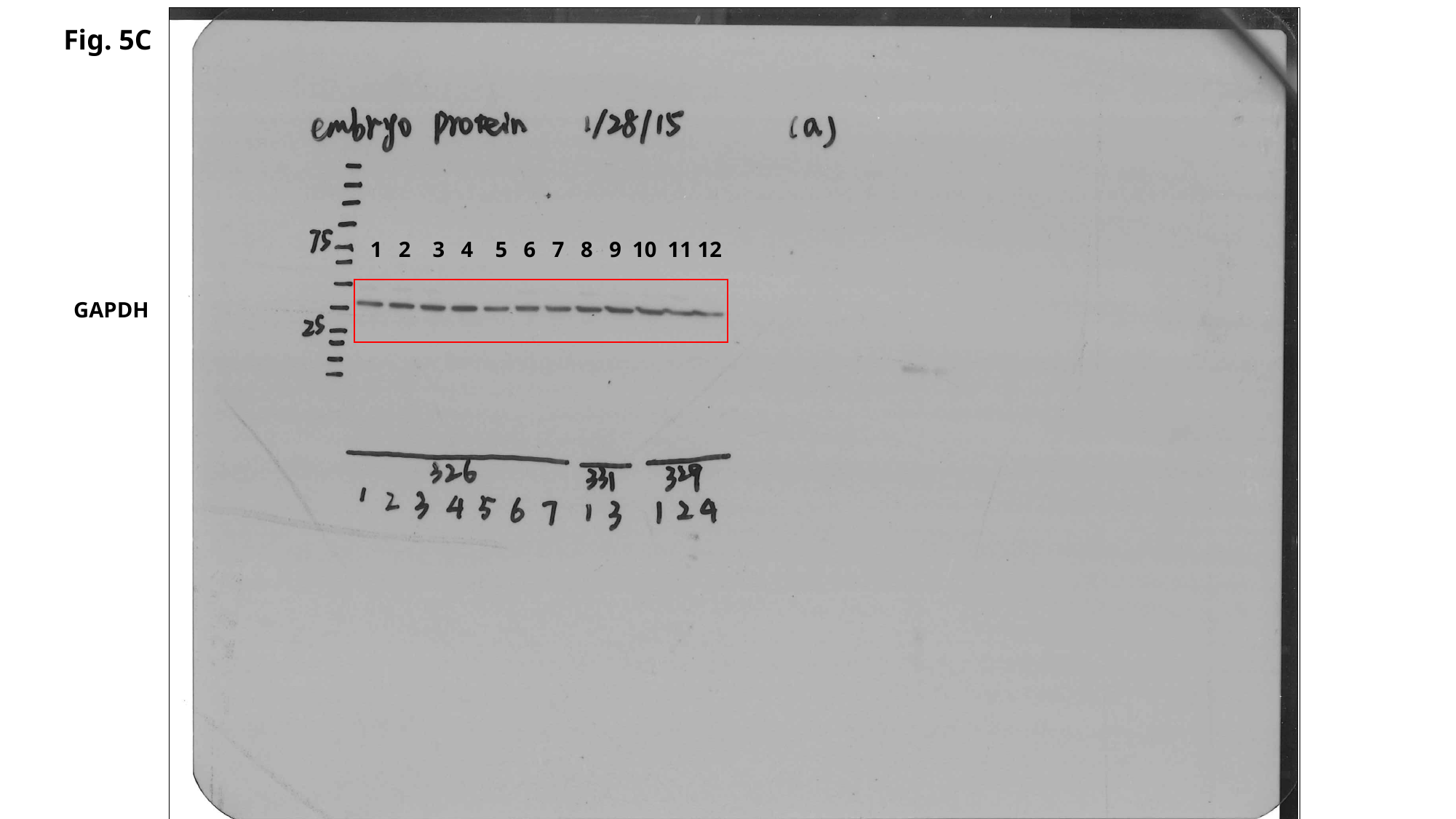

Fig. 5C
1 2 3 4 5 6 7 8 9 10 11 12
GAPDH
