# Supplementary figures and images for: OLA1 promotes colorectal cancer tumorigenesis by activation of HIF1α/CA9 axis
Source: BMC Cancer. 2022 Apr 19;22:424. doi: 10.1186/s12885-022-09508-1 (PMC9020043; doi:10.1186/s12885-022-09508-1)

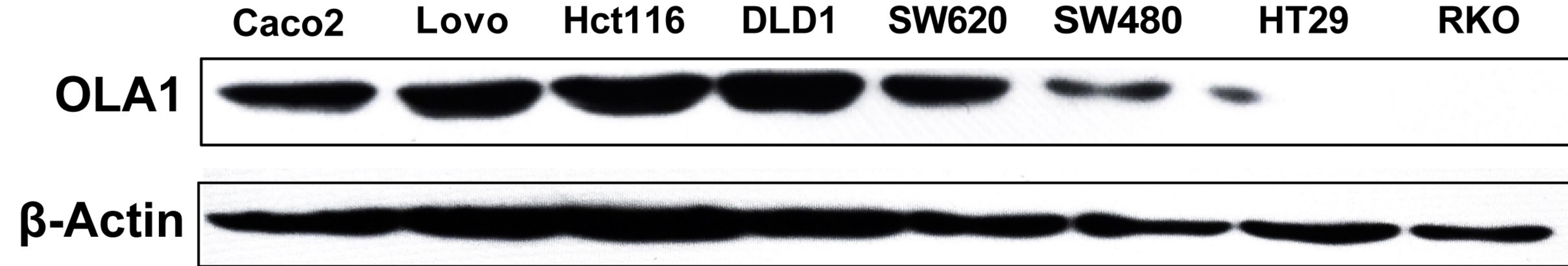

Figure S1 OLA1 expression in different colorectal cancer cell lines.

Supplement: Supplementary file 1 — Additional file 1. [file 12885_2022_9508_MOESM1_ESM.zip › Fig S1.pdf]
